# Investigating the Dietary Impact on Trans-Vaccenic Acid (Trans-C18:1 n-7) and Other Beneficial Fatty Acids in Breast Milk and Infant Formulas

**DOI:** 10.3390/foods13142164

**Published:** 2024-07-09

**Authors:** Laura Sanjulian, Alexandre Lamas, Rocío Barreiro, Ismael Martínez, Leopoldo García-Alonso, Alberto Cepeda, Cristina Fente, Patricia Regal

**Affiliations:** 1 Department of Analytical Chemistry, Nutrition and Bromatology, Faculty of Veterinary Medicine, Universidade de Santiago de Compostela, 27002 Lugo, Spain; laura.sanjulian.fernandez@usc.es (L.S.); alexandre.lamas@usc.es (A.L.); rocio.barreiro@usc.es (R.B.); alberto.cepeda@usc.es (A.C.); patricia.regal@usc.es (P.R.); 2 Feiraco Sociedade Cooperativa Galega, Ponte Maceira s/n, 15864 Ames, Spain; ismael.martinez@clun.es; 3 Pediatric Gastroenterology Unit, 15106 Corunna, Spain; dr.garcia.alonso@gmail.com

**Keywords:** breast milk, human milk, SEAD, Atlantic diet, trans-vaccenic acid, trans fatty acid

## Abstract

Maternal diet plays a significant role in the fatty acid composition of breast milk. Dietary products such as milk and meat are the primary sources of natural TFAs for humans. These peculiar fatty acids hold nutritional significance as they not only lack the detrimental effects of industrially produced *trans* fats on the endothelium characteristic, but they also exhibit anti-inflammatory properties. The relationship between the presence of eight fatty acids in breast milk (including natural TFAs *trans*-vaccenic and conjugated linoleic acid) and the maternal diet has been explored, and their abundance has been compared to that of infant formulas. Two cohorts of lactating women, originating from a Spanish region, participated in this study; they adhered to the Southern European Atlantic diet or the Atlantic diet. While the consumption of conventional meat or dairy products does not seem to increase the abundance of *TFAs* in breast milk, *trans*-vaccenic and oleic acid are among the most distinctive features of breast milk fat in mothers consuming naturally improved dairy products with an improved fatty acid profile. The most significant differences between natural breastfeeding and formula feeding lie in natural TFAs, since formulas are notably deficient in natural TFAs while being overfortified in alpha-linolenic acid in comparison to breast milk. We suggest an improvement in the formulation of these products through using cow’s milk with an optimal fatty acid profile that better mimics the fatty acid composition found in human milk.

## 1. Introduction

Breastfeeding is a biological process that protects neonates from malnutrition and even infectious diseases. The health benefits of breastfeeding for the mother and the baby are substantial and widely recognized worldwide. Human milk has been described as the gold standard of infant nutrition and serves as a valuable reference for establishing optimal nutrient intakes for infants who are unable to exclusively breastfeed [1]. For this reason, the composition of infant formulas should closely resemble that of breast milk [2]. The fatty acids (FAs) found in breast milk have been attributed to two distinct origins: (a) the release of fatty acids from external sources and (b) the de novo synthesis of fatty acids in the liver or mammary tissue. In this sense, a previous study by Barreiro et al. has underscored the significant impact of maternal diet on the fatty acid composition of breast milk [3].

Trans fatty acids (TFAs) cannot be synthesized de novo by the human body, so their exclusive sources are dietary intake [4] TFAs. TFAs such as elaidic acid (trans-9-C18:1) are typical of industrially hydrogenated vegetable fats and are often present in large quantities in commercial foods such as margarines and cakes [5]. They promote endothelial dysfunction and appear to negatively affect health by increasing the risk of disease for the circulatory system and type 2 diabetes [6]. These TFAs can also have a harmful effect on the development of babies, leading to lower levels of certain FAs which are important for normal brain and vision development, such as arachidonic acid (AA) and docosahexaenoic acid (DHA) [7]. Other types of TFAs can be naturally found in some foods. For example, natural TFAs are generated by the biohydrogenation of unsaturated FAs in the rumen of animals by bacterial isomerases and are therefore found in the milk, dairy products, and meat of these ruminant animals. The dominant TFAs from natural food sources are *trans*-vaccenic acid (TVA) (*trans*-C18:1 n-7), *trans*-palmitoleic acid (TPA), also known as *trans*-C16:1 n-7 or *trans*-9 C16:1, and conjugated linoleic acid (CLA) (main isomer—rumenic acid, *cis*-9, *trans*-11 C18:2). These natural TFAs have nutritional significance. Not only they do not seem to have a dysfunctional effect on the endothelium, they also exhibit anti-inflammatory properties [8]. In 2023 [9], it was reported that one of those natural TFAs, dietary TVA, could promote cytotoxic and tumor-infiltrating functions of effector CD8+ T cells, leading to greater in vivo antitumor immunity. In [9], through a comprehensive evaluation of this nutrient that is derived from the diet, progress was made in the identification and understanding of the mechanistic links between the diet and human physiology and pathology. Also, TPA has been recognized in prior epidemiological prospective studies as a biomarker that is indicative of metabolic health [10,11].

Dietary products, such as milk and meat, are the main sources of TFAs for humans, and they are derived from rumen fermentation. Intake of these fatty acids is exclusively ensured by ruminant-derived foods, such as milk, dairy products, and meat [12]. TVA is the major trans-C18:1 isomer in ruminant dairy fat [13]. The findings of Guillocheau et al. suggest that endogenous 16:1 t9 TPA is not exclusively derived—as has been previously assumed—from the diet; it may also be produced by the partial β-oxidation of dietary TVA [12]. In particular, the dietary patterns of the populations of northern Portugal and Galicia (northwest Spain), known as the Southern European Atlantic diet (SEAD) or the Atlantic diet (AD), are characterized by a high consumption of milk and dairy products and a moderate consumption of meat [14,15,16].

The FAs secreted in human milk have different origins: de novo synthesis in the breast and the maternal diet or body stores [17]. Additionally, FAs from the maternal diet can interact directly or indirectly with some transcription factors that would eventually produce changes in the composition of milk [7]. The scientific community agrees that the human body cannot synthesize TFAs, although some studies indicate that vaccenic acid can be converted to rumenic acid by Δ9-desaturase in the maternal gland during lactation [18]. Therefore, the TFA composition of breast milk is predominantly affected by the maternal diet [19]. Higher levels of the ruminal CLA isomer and its precursor TVA in breast milk correlate with lower rates of atopic manifestations in children. Perhaps this is because these natural TFAs modulate the immune function in the human body by reducing the production of pro-inflammatory mediators [20,21].

In previous studies with Galician women who were lactating, it was found that many of them adhere to the Atlantic diet, and factors such as maternal diet and prolonged lactation influence the lipid profile of human milk [22,23]. In the present work, we will discuss the relationship between the presence of eight natural FAs (including the natural TFAs: TVA and CLA), the adherence of Galician mothers to the healthy SEAD pattern, and the consumption of milk and dairy products (conventional or from improved production) and bovine meat, in addition to other factors, such as the duration of lactation. A comparison of breast milk with the FA levels of infant formulas that are available on the Spanish market is also included.

## 2. Materials and Methods

### 2.1. Samples

This is an observational cross-sectional design study, involving lactating mothers residing in the northwest of Spain (Galicia). The population in this region adheres to a dietary pattern known as the SEAD, characterized by the frequent consumption of milk, dairy products, and beef [14].

- Cohort 1: Women consuming conventional dairy products.

Milk sampling and data collection were carried out using convenience sampling in collaboration with the midwifery service and a local breastfeeding association. The study was registered on ClinicalTrials.gov under the following identification number: NCT03245697. Ethical approval for this study was granted by the Clinical Research Ethics Committee of Galicia (approval code 2016/280), and it adhered to the principles of the 1975 Declaration of Helsinki, revised in 1983. Informed written consent was obtained from all participants. Mothers collected human milk samples at home, extracting approximately 25 mL of milk manually or using a breast pump before breastfeeding their children. The samples were collected in sterile plastic tubes, stored between 3 and 5 °C, and delivered to the laboratory within 12 h; here, they were preserved at 24 °C. All samples were collected before the first morning feed or 2 h after the last session when the child breastfed at night [12]. Breast milk samples from 93 lactating women were utilized. Mothers were interviewed and preselected based on the following exclusion criteria: presence of acute or chronic illnesses; presence of metabolic disorders; gestation of less than 36 weeks; engaging in substance misuse (drugs or alcohol). All babies were healthy and growing well. Lactating women were surveyed for personal data, including age, medications, tobacco use, anthropometric data (body weight, height, and BMI), childbirth (delivery date, natural or cesarean section, and gender), and breastfeeding (duration in months after childbirth). Additionally, mothers were asked about their dietary habits (dairies and bovine meat foods consumption). Furthermore, a 9-item brief questionnaire (SEAD) was used to assess adherence to the diet [16,24]. None of the mothers were taking dietary supplements like vitamins, iron, or fish oil at the time of sample collection.

- Cohort 2: Women consuming improved dairy products.

The study incorporated breast milk samples from 16 mothers who consumed dairy products from improved milk, as part of a project approved by the Clinical Research Ethics Committee of Galicia (approval code 2010/027). The samples were collected from these women once month between 1 and 5 months postpartum, resulting in a total of 66 milk samples from the 16 participants, with colostrum being excluded. The volunteers collected the milk via manual extraction, and the samples were stored at −24 °C until further analysis. Throughout lactation, the mothers consumed an average of 3 daily servings of milk, yoghurt, and butter from a premium brand of UHT milk produced in Galicia called UNICLA. The diet of UNICLA dairy cattle includes a significant quantity of flax seeds, which is a source of unsaturated fatty acids such as omega-3, and selenized yeast, as a source of organic selenium [25]. An overview of UNICLA^®^ milk composition and differential comparison to conventional milk can be found in the literature [26].

Each participant underwent a face-to-face interview. Exclusion criteria were applied, including restrictions on diet, food intolerances, vegetarian or vegan diets, and non-consumption of major food groups outlined in the SEAD (fruits and vegetables, meat, eggs, milk and dairy products, fish, and cereals). The participating mothers were in good health, with no history of alcohol or substance misuse; they were non-smokers; they had no obstetric complications during pregnancy or delivery. None of the mothers were taking dietary supplements like vitamins, iron, or fish oil at the time of sample collection.

- Infant formula.

Twenty-three infant formulas were collected in different commercial areas and pharmacies of Spain, including five first-stage formulas (intended for babies from 0 to 6 months of age), thirteen follow-on infant formulas (intended for babies aged 6 months and above), and five growing-up milks (intended for young children). The samples were stored in the dark at −24 °C until analysis. The powdered formulas were reconstituted in water to obtain liquid formulas, following the manufacturer’s instructions, prior to analysis.

### 2.2. Fatty Acid Determination

The qualitative and quantitative determination of the fatty acid composition of breast milk was conducted using the method described by Sanjulián et al. [27]. Briefly, 10 uL of breast milk was digested overnight at 4 °C with acidified methanol, and fatty acids were subsequently methylated in a water bath for 2 h at 60 °C. Fatty acid methyl esters (FAMEs) were extracted from the previous solution with *n*-hexane and analyzed using a gas chromatography system (Agilent Technologies 6850, Santa Clara, CA, USA) equipped with a flame ionization detector (GC-FID) in Palo Alto, CA, USA. Fatty acid profiles of infant formulas were determined using the same methodology, which was applied to the reconstituted samples.

The total fat content of the breast milk samples was determined by mid-infrared spectroscopy. The total fat content of the infant formula samples was collected from their labelling.

### 2.3. Statistics

GraphPad^®^ Prism 10 version 10.2.2 (GraphPad Software, LCC, Dotmatics, UK) was used for statistical analysis and graphical representation. An unpaired t-test was used to compare the mean values of the fatty acids of two independent groups, constructed according to lactation time, maternal BMI, the gender of the infant, and/or maternal milk consumption, meat consumption, and adherence to the Atlantic diet. This test was also used to determine the differences in fatty acids between the two cohorts of mothers. To determine whether there were differences in fatty acids between the three groups of samples (two mother cohorts and the infant formulas), a one-way ANOVA was used with a post hoc Dunnet test to identify the pairs with significant differences. A *p* value < 0.05 was used to indicate statistically significant differences, meaning that the probability that any observed difference between groups is due to chance is 5%. Principal component analyses (PCAs) using a standardized method were carried out using data from the two cohorts only, and also including the cohorts and infant formulas.

## 3. Results and Discussion

### 3.1. Samples Data

#### 3.1.1. TFA, Diet, and Lactation Characteristics

- Cohort 1

A cohort of lactating women, belonging to a cross-sectional observational study that was designed to investigate the composition of the breast milk of lactating mothers residing in the northwest of Spain (Galicia), has been studied. Ninety-three lactating women, aged between 26 and 46 years, were recruited; they generously donated their milk samples. The samples were collected between 1 and 59 months postpartum [14,24]. All the mothers and children were healthy; only 9 women (12%) underwent a C-section delivery and 60% were primiparous. Data on gestation and on maternal and newborn characteristics are provided in Table 1. Continuous data are expressed as mean and standard deviation (SD). Categorical variables are expressed as percentages.

- Cohort 2

Sixteen lactating women, aged between 26 and 35 years, were recruited; they generously donated their milk samples. The samples were collected between 1 and 5 months postpartum, with 13 women providing samples at month 4 and only 3 providing samples at month 5.

The participants in cohort 2 consumed at least three servings per day of a type of premium milk produced in Galicia, UNICLA^®^.

#### 3.1.2. TFA across Samples

A total of eight fatty acids were analyzed in all the samples: oleic acid (OA), linoleic acid (LA), alfa-linolenic acid (ALA), arachidonic acid (AA), eicosapentaenoic acid (EPA), docosahexaenoic acid (DHA), trans-vaccenic acid (TVA), and CLA (cis-9,trans-11 CLA and trans-10,cis-12 CLA isomers). Table 2 displays the mean levels of each fatty acid across the different groups of samples, including the groups according to the lactation time (LT), the body mass index (BMI), the newborns’ sex, the SEAD score, the milk and meat consumption, and the consumption of dairy products that were naturally enriched in ruminal trans FAs. Table 2 also includes the mean levels of fatty acids in each group of infant formulas (first stage formula—FSF; follow-on formula—FOF; growing up formula—GUF).

The total fat contents in the analyzed breast milk samples ranged between 2.6 and 4.2 g/100 mL. The declared content of total fat in the analyzed formulas ranged between 2.5 and 4.1 g/100 mL.

- Cohort 1

In the production of breast milk, some compounds, such as CLA or TVA, partly come from the mother’s diet, while others are produced by the mother’s body. Dietary products from ruminants (milk and meat) are the main sources of natural TFAs for human consumers [26]. Several studies have examined the effect of CLA supplementation on the presence of natural trans FAs in breast milk, but little is known about their short-term transfer. Recent data suggest that concentrations of CLAs and TVAs in breast milk can be influenced by the diet. The intake of meat and meat products was associated with higher levels of vaccenic acid and total TFAs in human milk among lactating women in Latvia [19]. It is recommended that the source of these FAs in the diet of breastfeeding women be natural products rather than dietary supplements [19,27]. An analysis of data from cohort 1 showed that the FA fraction remained constant across the groups, with the exception of OA, which is the most abundant FA; for OA, significant differences were found based on maternal BMI (*p* = 0.0210) and among women consuming more than three servings of meat per week (*p* = 0.0157). Other authors have found a relationship between OA content and the nutritional status of mothers, specifically with undernutrition status. The statistical tests did not find significant differences (*p* > 0.05) between the levels of oleic acid among breastfeeding mothers with chronic energy deficiencies and those with normal nutritional statuses; however, in the study by Muhrifan [28], lower levels were found among women with chronic energy deficiencies. However, the consumption of conventional meat or dairy products does not appear to influence a higher abundance of TFAs in breast milk.

- Cohort 1 versus cohort 2

The milk consumed by cohort 2 is distinguished by its improved nutritional composition. This is achieved by modifying the cow’s diet by incorporating high-quality forages and improving digestibility, instead of artificially altering the final product. The main components of the diet of dairy cows in Galicia are grass and corn silage; farmers harvest, store, and supply these to their livestock throughout the year, along with forage produced by Feiraco S.L. (the cooperative of stockbreeders) as a supplement to improve the nutritional profile of the milk; thus, the composition of the forage was modified. The objective of this process is to provide a balanced and sufficient ration to dairy cattle, reproducing the fatty acid profile of spring pastures.

The approach to cow milk production can affect its composition; this has been studied extensively in the case of lipid composition. Several studies have shown that cow milk from animals which are fed on pastures or which are raised according to organic production guidelines [28,29,30] has higher contents of natural TFAs than conventional milk. The hypothesis that the amount of CLAs and TVAs in human milk could be increased by increasing the dietary amount of organic ruminant products has been studied in the Koala cohort [31]. In the milk of participants in the Koala cohort, the CLA content and its main precursor, TVA, increased when transitioning from a conventional diet to a moderately organic or strictly organic one. In a study by Simões-Wüst et al. [32], it was demonstrated that the rumenic acid content was higher in the breast milk of participants who consumed a type of organic dairy product called biodynamic compared to those consuming conventional or organic dairy products other than biodynamic, which was intermediate. Biodynamic organic farms consider—in addition to the organic production guidelines—cosmic rhythms, which they believe result in improvements in plant and livestock production (http://www.demeter.net/, accessed on 22 April 2024). In the case of TVA, a profile comparable to that of CLA was found. Since TVA can be converted into rumenic acid in humans [18,33], it seems logical that their values are parallel. Again, the highest level of TVA was detected to a greater extent in the milk of women who consumed biodynamic dairy products, followed by the group of women who consumed other organic dairy products and the group of conventional dairy products. Although these differences were not statistically significant, the authors argue that the physiological importance of these apparently moderate differences should not be underestimated. In fact, in the studied cohort, an inverse correlation was already demonstrated between the levels of CLA in the milk of participants and the presence of a pathological condition in their children (atopic diseases and eczema) [20].

In cohort 1 in the present study, samples were categorized into two groups based on lactation time (LT), as infants typically begin complementary feeding at 6 months. The early-lactation group (<6 months) was used for comparison with samples from cohort 2, which included participants who had been lactating for up to 5 months. The unpaired t-test results showed significant differences in the content of some fatty acids evaluated: the contents of OA (*p* < 0.0001), TVA (*p* < 0.0001), and CLA (*p* < 0.0055) are significantly higher in samples from cohort 2. This is consistent with the previous research findings explained above, that have linked the consumption of organic dairy products to an increase in the levels of these fatty acids in breast milk [31,32].

**Figure 1:**
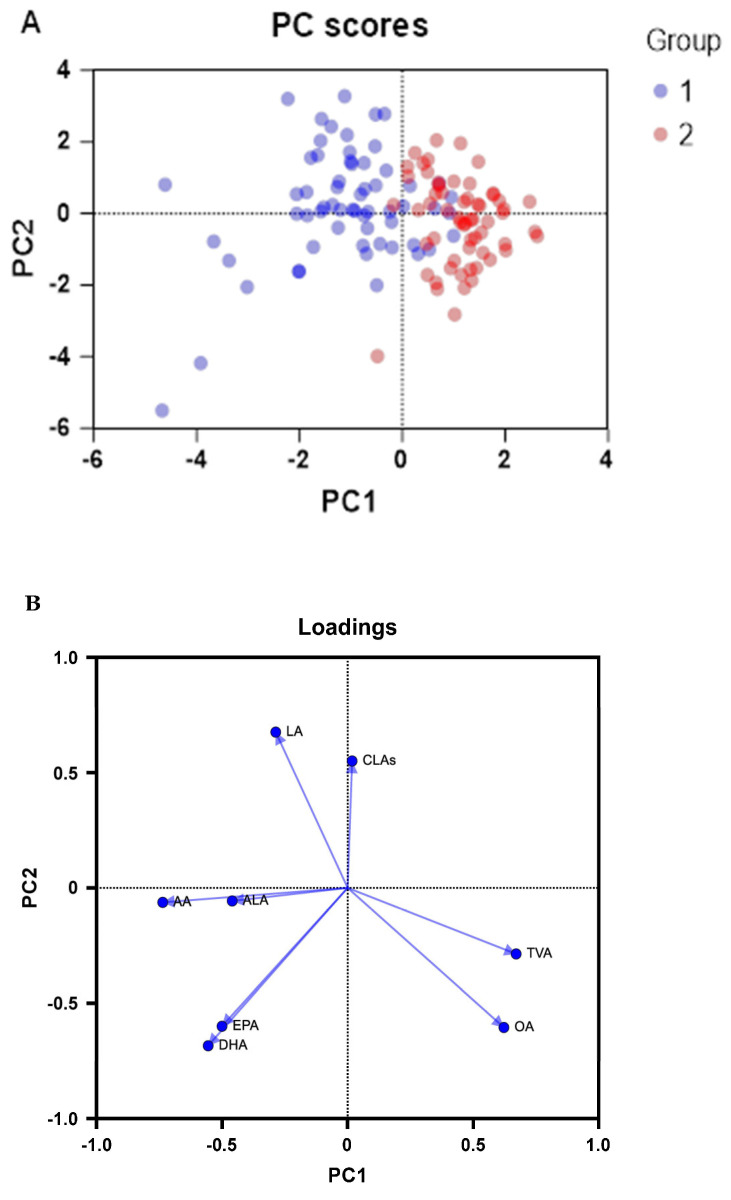
PCA score scatter plot (**A**) and loading plot (**B**) of fatty acid profiles of breast milk from cohorts 1 and 2.

Principal component analysis (PCA) is a statistical technique that is used to synthesize the data analyzed by reducing the dimensions. In other words, the number of original variables is reduced while losing a minimum amount of information. The principal components obtained are a linear combination of the original variables and are independent of each other. PCA, using a standardized method, was carried out including the two cohorts. Figure 1 shows a separation between the samples of cohort 1 and those of cohort 2. The loadings graph provides insight into the importance of the variables in the distribution of the samples in the principal components analysis graph.

A score scatter plot shows the existence of cohort-dependent features in the fatty acid profiles; the milk samples from the cohort 2 participants, who consumed CLA-enriched dairy products, are plotted relatively far from the milk samples from participants in cohort 1. In this sense, the loading plot shows that TVA and OA are among the most distinctive features of breast milk fats in cohort 2. Conversely, cohort 1 samples scored lower for these two fatty acids. An increased concentration of both TVAs in human milk was correlated with a lower risk of eczema, atopic dermatitis, and allergic sensitization in the infants at one year of age [34]. On the positive side of PC1, there is a large weighting for the TVA and OA values in the ranking of the samples. This indicates that the concentrations of these two fatty acids are of great importance in the separation of the two cohorts. On the other hand, on the negative side of PC1, we observe the load of EPA and DHA, as well as AA and ALA, in the grouping of the samples. This would indicate the specific fatty acid profiles and characteristics of each cohort.

- Breast milk vs. infant formula

In a previous study conducted by our research group [35], no significant differences were found in the fatty acid composition of formulas from different categories or stages, except for those enriched or fortified with specific fatty acids. In the same study, it was observed that, during the first 6 months of lactation, breast milk had less oleic acid; it was slightly more saturated than monounsaturated, and contained more LA, ALA, AA, and DHA than it did in the subsequent months of lactation (>6 months). It is during the first months of life that a baby requires a greater amount of these fatty acids due to neuronal and retinal development.

**Figure 2:**
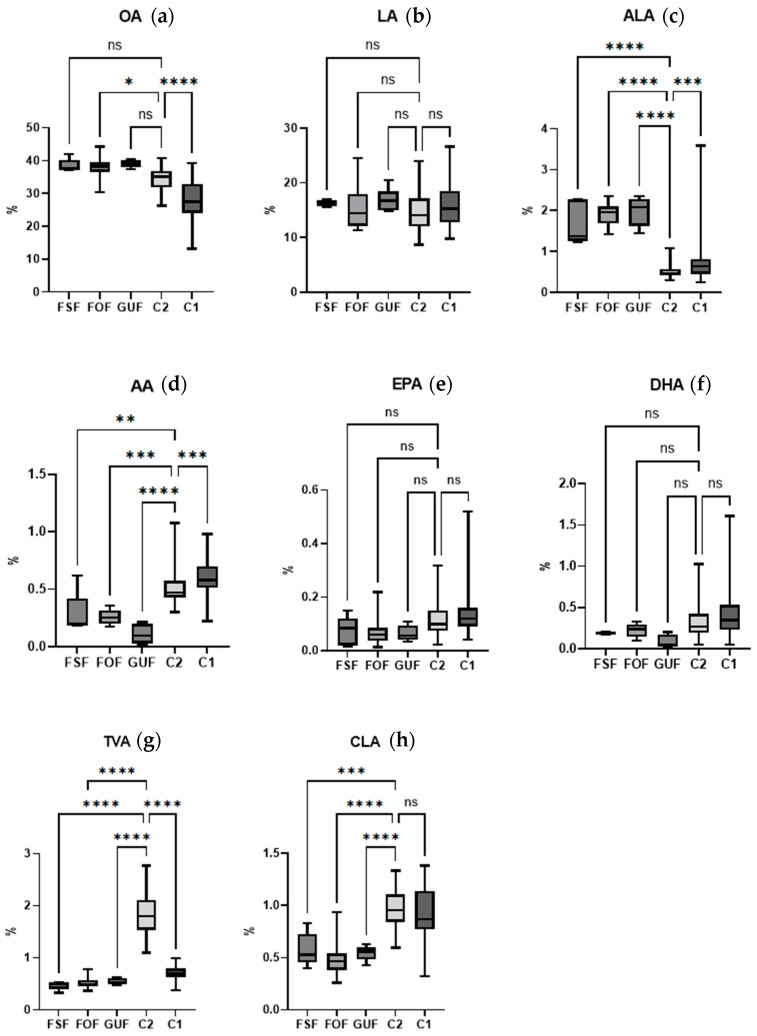
Box plots showing the abundance of OA (**a**), LA (**b**), ALA (**c**), AA (**d**), EPA (**e**), DHA (**f**), TVA (**g**), and CLA (**h**) fatty acids in breast milk samples from cohorts 1 and 2 (C1 < 6 months, C2) and in infant formula (first stage formula—FSF; follow-on formula—FOF; growing up formula—GUF). Significant differences between groups are highlighted with * (*p* < 0.05), ** (*p* < 0.01), *** (*p* < 0.001), or **** (*p* < 0.05). ns: no significant differences.

The fatty acid compositions of milk formulas marketed in Spain and human milk from the two cohorts of participants were compared using one-way ANOVA with a post hoc Dunnett test. For this test, only samples < 6 months of lactation were considered. Figure 2 shows the plots for each of the fatty acids studied.

The box plots show the distribution of each fatty acid value in the groups included in the study. The box represents the interquartile range (IQR), i.e., the range from the first quartile (minimum) to the third quartile (maximum). The whiskers extend up to 1.5 times the IQR, which allows the visualization of data dispersion and outliers. For example, in cohort 1, it is observed that, although most of the samples have similar percentages of ALA, DHA, and EPA (indicated by a small box), there is a significant dispersion in some values, given the long whisker length observed. This shows the individual variation in the breast milk itself.

The results observed in Figure 2 for TVA deserve special attention. The boxes for infant formula and cohort 1 show very homogeneous percentages in the samples forming these groups, all being below 1%. In the case of cohort 2, there is greater dispersion between samples, but the percentages are always above 1% and are significantly higher than those in the infant formula and in cohort 1.

The presence of OA is higher in all infant formulas, but the difference was not significant. ALA is a fatty acid with a significantly higher presence in all infant formulas studied than in maternal milk. In contrast, the content of AA is lower in infant formulas than in maternal milk. AA has very different biological functions compared to DHA; for example, there are different functions in the vasculature and the specific aspects of their immunity. DHA suppresses the concentrations of arachidonic acid in the membrane and suppresses its functions. An infant formula with DHA and without arachidonic acid is at risk of inducing cardiovascular and cerebrovascular morbidity and even mortality due to the suppression of favorable oxylipin derivatives of AA [36]. The differences were not significant for DHA and EPA; however, in all three types of formulas, the amount was lower than in the two breast milk cohorts. However, the greatest differences between natural and formula feeding are in the availability of natural TFAs; all the infant formulas analyzed in this study are clearly deficient in natural TFAs.

## 4. Conclusions

Infant formulas available in the Spanish market are found to contain significantly lower levels (all being below 1%) of natural TFAs compared to breast milk. These fatty acids, such as trans-vaccenic acid (TVA) and conjugated linoleic acid (CLA), play a critical role in infant health. Mothers who integrate high-quality dairy products into their diets exhibit increased levels of TVA and CLA in their breast milk (always above 1%), potentially leading to enhanced health outcomes for their infants. Given the deficiency of natural TFAs in infant formulas, we suggest emphasizing the formulation of these products with cows’ milk that has an optimal fatty acid profile which better mimics the fatty acid compositions that are found in human milk.

## Figures and Tables

**Table 1:** Pregnancy and maternal characteristics. Continuous and categorical variables (*n* = 92).

Maternal Data	Mean	Median	Min	Max	
Pregnancy Time (weeks)	39.77 ± 1.34	40.00	36.00	42.29	
Maternal Age (years)	35.43 ± 4.06	35.00	26.00	46.00	
Maternal BMI (kg/m^2^)	24.47 ± 3.83	24.36	17.85	35.03	
Lactating time (months)	7.77 ± 11.12	3.00	0.5	58.97	
AD adherence (score)	3.80 ± 1.48	4.00	0.00	7.00	
Infant gender: *n*♂ (%)/*n*♀ (%)	43 (46.73)/49 (53.26)	C-section delivery (%)	11.96	Parity number 1st child (%)	60.87

**Table 2:** Oleic acid (OA), linoleic acid (LA), α-Linolenic acid (ALA), arachidonic acid (AA), eicosapentaenoic acid (EPA), docosahexaenoic acid (DHA), trans-vaccenic acid (TVA), and conjugated linoleic acid (CLA, sum of cis-9,trans-11 CLA and trans-10,cis-12 CLA isomers), presented as mean, standard deviation, and range across different groups of breast milk samples and infant formula (first stage formula—FSF; follow-on formula—FOF; growing-up formula—GUF). Variables considered are lactating time (LT, months), body mass index (BMI), newborn sex (0 = boy, 1 = girl), SEAD adherence (score), milk and meat consumption (servings per week), naturally enriched dairy products consumption (cohort 1—no; cohort 2—yes).

			Cohort 1											Cohort 2	Infant Formula		
Fatty Acid (%wt/wt of Total Fatty Acids)		LT < 6	LT ≥ 6	BMI < 25	BMI ≥ 25	Infant 0	Infant 1	SEAD < 5	SEAD ≥ 5	Milk < 3	Milk ≥ 3	Meat < 3	Meat ≥ 3	LT < 6	FSF	FOF	GUF
		*n* = 67	*n* = 26	*n* = 47	*n* = 46	*n* = 41	*n* = 52	*n* = 64	*n* = 29	*n* = 39	*n* = 41	*n* = 63	*n* = 18	*n* = 16	*n* = 5	*n* = 13	*n* = 5
18:1 (n-9) OA	Mean	28.35 *	29.38	**30.02**	27.34	30.12	27.47	28.17	29.67	28.41	29.49	**27.74**	31.88	34.49 *	38.49	38.06 *	39.07
	SD	5.87	8.37	6.85	6.20	6.81	6.30	6.86	6.05	6.96	6.00	6.20	6.58	3.22	2.06	3.44	1.18
	Min	13.11	14.52	14.52	13.11	19.76	13.11	13.11	20.03	13.11	20.15	13.11	22.50	26.21	37.10	36.45	37.32
	Max	39.28	43.19	42.57	43.19	43.19	41.58	43.19	42.11	42.11	43.19	43.19	42.11	40.67	42.04	44.20	40.43
18:2 (n-6) LA	Mean	15.96	15.78	15.63	16.18	15.68	16.10	15.84	16.08	16.64	15.23	16.50	14.53	14.60	16.26	15.64	16.96
	SD	3.84	4.17	4.13	3.73	4.03	3.85	3.77	4.27	4.10	3.85	3.99	2.44	3.41	0.58	3.91	2.32
	Min	9.75	8.73	8.73	9.42	8.73	9.75	8.73	9.42	9.42	8.73	8.73	9.42	8.66	15.50	11.54	14.90
	Max	26.64	23.86	26.64	24.80	24.80	26.64	24.80	26.64	26.64	22.68	26.64	19.62	23.97	16.94	24.53	20.51
18:3 (n-3) ALA	Mean	0.75 *	0.91	0.91	0.68	0.66	0.89	0.83	0.70	0.94	0.71	0.79	0.70	0.50 *	1.69 *	1.88 *	1.98 *
	SD	0.49	0.75	0.74	0.33	0.29	0.71	0.66	0.31	0.69	0.37	0.52	0.29	0.13	0.54	0.28	0.36
	Min	0.25	0.31	0.31	0.25	0.25	0.31	0.31	0.25	0.31	0.25	0.25	0.40	0.30	1.23	1.69	1.45
	Max	3.59	4.12	4.12	1.69	1.55	4.12	4.12	1.54	4.12	1.67	3.59	1.54	1.08	2.27	2.35	2.35
20:4 (n-6) AA	Mean	0.60 *	0.60	0.58	0.61	0.59	0.60	0.59	0.61	0.58	0.60	0.59	0.56	0.45 *	0.28 *	0.26 *	0.11 *
	SD	0.14	0.19	0.16	0.16	0.16	0.15	0.16	0.15	0.15	0.18	0.15	0.15	0.11	0.19	0.07	0.09
	Min	0.22	0.30	0.30	0.22	0.22	0.31	0.30	0.22	0.30	0.22	0.22	0.35	0.28	0.18	0.28	0.02
	Max	0.98	1.00	0.98	1.00	1.00	0.98	1.00	0.98	1.00	0.98	1.00	0.88	0.72	0.62	0.36	0.21
20:5 (n-3) EPA	Mean	0.14	0.12	0.15	0.13	0.12	0.15	0.14	0.13	0.14	0.15	0.14	0.10	0.11	0.07	0.07	0.06
	SD	0.09	0.08	0.09	0.09	0.08	0.09	0.09	0.09	0.07	0.12	0.08	0.05	0.06	0.05	0.05	0.03
	Min	0.04	0.05	0.06	0.04	0.04	0.05	0.05	0.04	0.05	0.04	0.04	0.05	0.02	0.02	0.01	0.03
	Max	0.52	0.35	0.51	0.52	0.52	0.51	0.52	0.51	0.35	0.52	0.52	0.29	0.32	0.15	0.22	0.11
22:6 (n-3) DHA	Mean	0.42	0.50	0.48	0.40	0.39	0.48	0.44	0.43	0.44	0.47	0.41	0.37	0.32	0.21	0.22	0.09
	SD	0.32	0.30	0.33	0.29	0.27	0.34	0.30	0.34	0.28	0.37	0.29	0.24	0.18	0.05	0.08	0.08
	Min	0.05	0.17	0.05	0.05	0.05	0.05	0.05	0.05	0.05	0.05	0.05	0.05	0.05	0.18	0.28	0.01
	Max	1.61	1.23	1.61	1.41	1.41	1.61	1.41	1.61	1.23	1.61	1.41	0.96	1.03	0.30	0.33	0.20
18:1 (n-7) TVA	Mean	0.71 *	0.69	0.70	0.71	0.70	0.71	0.71	0.70	0.69	0.75	0.70	0.72	1.84 *	0.47 *	0.53 *	0.54 *
	SD	0.13	0.13	0.12	0.13	0.14	0.12	0.12	0.14	0.12	0.15	0.12	0.14	0.41	0.08	0.11	0.06
	Min	0.38	0.41	0.43	0.38	0.38	0.42	0.42	0.38	0.41	0.38	0.38	0.41	1.10	0.33	0.45	0.48
	Max	0.99	0.87	0.99	0.94	0.95	0.99	0.95	0.99	0.94	0.99	0.95	0.94	2.77	0.53	0.79	0.63
CLAs	Mean	0.93	0.86	0.89	0.93	0.88	0.93	0.93	0.85	0.96	0.85	0.94	0.85	0.97 *	0.58 *	0.50 *	0.54 *
	SD	0.23	0.30	0.26	0.24	0.26	0.24	0.25	0.24	0.26	0.25	0.26	0.23	0.19	0.16	0.19	0.07
	Min	0.32	0.50	0.41	0.32	0.32	0.59	0.41	0.32	0.41	0.32	0.32	0.57	0.60	0.40	0.42	0.43
	Max	1.38	1.42	1.42	1.38	1.42	1.38	1.42	1.31	1.42	1.29	1.42	1.33	1.33	0.83	0.94	0.63

*n*—number of samples; numbers in bold indicate significant differences (*p* < 0.05) between two groups of comparison in cohort 1; significant differences between cohort 2 and cohort 1 (LT < 6), and between cohort 2 and different formulas, are highlighted with an asterisk.

## Data Availability

The original contributions presented in the study are included in the article, further inquiries can be directed to the corresponding author.
